# Lessons Learned from Implementing the Patient-Centered Medical Home

**DOI:** 10.1155/2012/103685

**Published:** 2012-08-30

**Authors:** Ellen P. Green, John Wendland, M. Colette Carver, Cortney Hughes Rinker, Seong K. Mun

**Affiliations:** ^1^University of Delaware, 416C Purnell Hall, Newark, DE 19711, USA; ^2^Carilion Clinic, Roanoke, VA 24014, USA; ^3^George Mason University, Fairfax, VA 22030, USA; ^4^Arlington Innovation Center for Health Research, Virginia Tech, National Capital Region, Arlington, VA 22203, USA

## Abstract

The Patient-Centered Medical Home (PCMH) is a primary care model that provides coordinated and comprehensive care to patients to improve health outcomes. This paper addresses practical issues that arise when transitioning a traditional primary care practice into a PCMH recognized by the National Committee for Quality Assurance (NCQA). Individual organizations' experiences with this transition were gathered at a PCMH workshop in Alexandria, Virginia in June 2010. An analysis of their experiences has been used along with a literature review to reveal common challenges that must be addressed in ways that are responsive to the practice and patients' needs. These are: NCQA guidance, promoting provider buy-in, leveraging electronic medical records, changing office culture, and realigning workspace in the practice to accommodate services needed to carry out the intent of PCMH. The NCQA provides a set of standards for implementing the PCMH model, but these standards lack many specifics that will be relied on in location situations. While many researchers and providers have made critiques, we see this vagueness as allowing for greater flexibility in how a practice implements PCMH.

## 1. Introduction

In response to the increasing demand for an improved healthcare system in the United States, the American Academy of Family Physicians, the American Academy of Pediatrics, the American College of Physicians, and the American Osteopathic Association developed the Joint Principles of the Patient-Centered Medical Home (Table 1 [1]) [2]. The Patient-Centered Medical Home (PCMH) is an extension of internationally employed Edward Wagner's Chronic Care Model (CCM). The CCM was developed to address the increasing rate of patients with chronic conditions in the United States using team-based care. The rate of chronic conditions is currently estimated to be 2.2 conditions for individuals having 60 years old and up, on average [3]. In its implementation, the CCM has proven to reduce patients' healthcare costs and improve patient care quality, two elements directly aligned with the goals of the PCMH [3]. The PCMH model strives to provide quality, coordinated, and cost-effective care to patients and to increase access to services. In addition, it aims to increase practice efficiency and subsequently provider and patient satisfaction. Within this paper, we follow the process of implementing the PCMH Model within primary care practices and discuss the difficulties these practices have encountered in the transition as well as potential solutions. Our goal is to provide future PCMHs with an insight into the transition process and to remove the likelihood of these problems reoccurring. 

In spite of difficulty with the transition process, over 1,500 sites and 7,700 clinicians across the United States have successfully completed the National Committee for Quality Assurance (NCQA) recognition process and are functioning as Patient-Centered Medical Homes [4]. They see PCMH as a way to better serve their patients, to address the crisis in primary care, and to seize evolving payment opportunities. Encouraged by the expansion of the PCMH across the United States, in June 2010, medical practitioners and healthcare administrators (military providers, civilian doctors and nurses, researchers, hospital staff, and administration) met in Alexandria, Virginia, to discuss their experiences with the transition. From the conference, we were able to collect detailed experiences that will provide a unique insight and data from the transition process. From the panel of attendees, two specific healthcare providers—Carillion Clinic and the Air Force—contributed the vast majority of the examples and experiences discussed here. 

Carillion Clinic is a large healthcare organization located in Southwest Virginia, providing healthcare to individuals in both urban and rural settings. Their organization comprises over 600 physicians in multicare-specialty group practices and eight not-for-profit hospitals [5]. The examples in this paper are primarily taken from their experiences transitioning their urban primary care practices into PCMHs. More specifically, their experiences were directly pulled from practices with multiple physicians (Carilion Clinic does group certifications of PCMHs by region). 

The Air Force has employed their version of the PCMH, termed the Air Force Patient-Centered Medical Home (previously the Family Health Initiative), at several of their bases within the United States. The Air Force PCMH was modeled after the qualities and goals of a PCMH. These Air Force PCMH practices are within the Air Force Bases themselves, and each of them has a patient panel of military beneficiaries (active duty members, retirees, and families) creating a unique healthcare environment because the Air Force operates under a complex healthcare and insurance system with both military care and purchased care (Our focus in this paper is on the onbase care provided by the Air Force and does not pertain to care purchased offbase through the Air Force's TRICARE program). 

From these healthcare organizations' experiences, combined with a literature review of empirical work, this paper addresses the challenges and successes encountered in the transition to PCMH. The purpose of this paper is to realize the difficulties that arise in the transition into a PCMH across various settings and to provide solutions for practices to follow in their transition given their particular needs. This way these practices can provide better quality of care to patients, decrease patient's health care costs, and improve the system as a whole. The paper is as follows, after a discussion of NCQA recognition, “Lessons Learned” discusses the most common concerns among primary care practices who have initiated the transition to a PCMH, which are promoting physician buy-in, changing office culture, care coordination, staffing and space allocations, and leveraging electronic medical records (EMRs). Under each of these topics, difficulties and successes are examined using specific examples provided by the PCMH workshop and previous studies. 

## 2. NCQA Recognition 

The first step in the transition process is to understand what procedures and standards a primary care practice must follow in order to obtain recognition. Recognition as a PCMH increases the likelihood of reimbursement for the pioneering PCMH procedures that are currently undercompensated. As more studies discuss the positive results of PCMH, new reimbursement methods become more of an obtainable goal. For a practice to become recognized as a PCMH by the NCQA, the practice must provide documentation of the practice's guidelines for implementation (Table 2, [6]). However, NCQA standards do not offer instructions for practices to follow in making the transition. This flexibility in the NCQA standards is necessary due to the uniqueness of each practice as it would be difficult to create one that addresses the implementation of all the standards of the PCMH for each practice type. Consequently, each practice must create its own written policies that adhere to PCMH principles and fit the specific practice structure. Practices must account for their patient panels, location, and financial resources when creating policies. The practices must then decide whether it would better suit their needs to attempt the transformation incrementally or all at once [7]. Once these policies have been written, implemented, and then the outcomes documented, the practice can apply for NCQA recognition through an online survey that collects information regarding its guidelines about administration (appointments, access, telephone calls), clinical services (patient satisfaction, tracking critically important conditions), and performance tracking [8, 9]. Once these steps are completed successfully as determined by the NCQA review process, the practice is awarded one of three tiers of recognition by the NCQA (The NCQA is currently debating reframing the recognition system to a two-tiered recognition system. Some supporters of the PCMH feel that the implementation of the model should be to the fullest extent possible and only acknowledged in by the NCQA in these cases. However, as discussed within this paper, there are difficulties that arise when attempting to administer all of the changes necessary and not all practices can achieve the standards required for these recognition levels). Each tier reflects how many performance elements under each NCQA standard the practice has satisfied. As with the introduction of any innovative healthcare model, the transition and recognition of a PCMH can be time consuming and expensive, but rewards range from better patient care to a more coordinated practice structure and also potentially reducing the overall cost of care for the patient in their lifetime through preventative and care management services [2], the latter of which is becoming more and more of a concern for primary care practices due to the current debate over reimbursement schedules.

## 3. Lessons Learned

### 3.1. Promoting Physician Buy-In

Primary care physicians typically leave the workforce earlier in their careers than specialists with complaints of being overworked and poorly compensated [10]. Therefore, selling the model to physicians, with their already hectic schedules, so that their participation and contribution to PCMH is pivotal in a successful transformation. However, many physicians involved in the transformation resist the change due to a lack of the appropriate PCMH training, misaligned financial incentives, underreimbursement, and time-consuming procedures [11]. 

#### 3.1.1. Training and Reimbursement Schedule

The PCMH model recommends that practice staff and providers (nurses and physicians) take time to analyze patients' needs as a whole. The model encourages practice leaders to empower ancillary staff to enlist protocols when meeting chronic, acute, and health maintenance needs. This team-based care is delivered prior to or after the provider encounter during a visit. The Air Force gave the following example of team-based collaboration when addressing a patient with a sprained ankle. First, the technician evaluates the patient and then performs the Ottawa ankle rules, which clearly delineate whether the patient requires an X-ray. Next, nurses will order X-rays based upon the results of the Ottawa ankle rules the technician provides. Subsequently, the skilled nurses return to educate the patient on ankle exercises and necessary bracing. Training a nurse to do this initial triage and intervention enables the provider to meet with the patient after X-rays are complete, which allows for a more efficient appointment. The team-based care creates a routine in which it is no longer necessary for a provider to address the patient each time they come to the practice. Many physicians are reluctant to release these face-to-face encounters with patients to their staff. This can be explained by the lack of training specific to PCMH practices, as well as the current reimbursement schedule, which encourage physicians to treat patients based on volume of care, rather than quality [11]. Practices must emphasize that the reallocation of responsibilities across the practice's medical team can create more time for them to complete other requirements and make the practice more efficient as seen in the above Air Force example. 

Second, the current form of financial reimbursement creates misaligned incentives for the physicians with their new responsibilities. For instance, according to the Medicare Resource-Based Relative Value Scale (RBRVS), physicians are only reimbursed for a patient visit if they can report a 15-minute office evaluation, discouraging the physicians from relinquishing any of their patients' visits to their nurses [12]. Restructuring the reimbursement scheme so that such face-to-face visits between physicians and patients are not required would allow physicians to provide care within the best interest of the patient and their practice. However, this modification may involve many hours of lobbying and petitioning the federal government and insurance companies. With many successful PCMHs across the United States, in order to sustain the model payment schemes will eventually need to adjust to reimburse physicians for improving patients' quality of care and overall health, rather than for just the volume of services they provide. Aside from physicians' reimbursement, other components of the PCMH policies, such as care coordination and team-based care, require the use of resources not currently reimbursed in most health insurance packages. This creates a sizeable draw on the primary care practice's monetary resources. 

### 3.2. Team-Based Care

In addition to the above-mentioned responsibilities of the physicians and their staff, the PCMH model introduces team-based care to primary care practices [13]. Team-based care requires staff to address patient treatment decisions collectively. In advance of each appointment, a previsit meeting is conducted to allocate resources, to ensure that adequate health maintenance is performed, and to see that chronic care management is addressed by the care team (nurses, care coordinators, physicians, other providers in the practice, and front office staff). Some staff see this as an unnecessary and cumbersome distraction from their routine [14]. However, it is important to emphasize that previous studies show the use of previsit huddles has resulted in improved decisionmaking and care for patients, as well as patient satisfaction [15, 16]. The studies also show that patients provided with team-based care had increases in their satisfaction levels from 5 to 10 points in five out of six satisfaction care scales. In addition, their research shows that once providers accepted the team-based care structure, their satisfaction levels increased as well.

In spite of the current difficulty with physician buy-in to PCMH, we found that many physicians are inspired by their ability to provide patients with the quality care they deserve through PCMH. A lead primary care physician from a Carillion Clinic has seen an improvement in the health of patients due to the coordination and team-based care PCMH offers

**The PCMH care model has helped us provide more comprehensive care to our patient population. The use of registries and care coordinators have enabled us to focus on patients that are not getting needed care or are not returning for follow-up appointments as they should.**

### 3.3. Changing Office Culture

The transformation of a primary care practice into a PCMH requires significant changes in office culture. In this section, we will discuss several areas that need to be addressed by the primary care practice, including nurse and patient experience. 

#### 3.3.1. Nurse and Technician Experience

Implementing the PCMH model is not as simple as assigning new staff roles and restructuring old staff with new titles. The healthcare organization must understand how changing positions within the practice brings new experiences to all of their current members and how these experiences can potentially affect their capability of delivering care [17]. These changes, if left unaddressed, can result in unfocused care. For instance, in the Air Force, many nurses in the PCMH transition shift from positions where they have not been exposed to patients to being required to evaluate patients face to face regularly; this new interaction created anxiety among these nurses. The Air Force has addressed this issue through additional training programs so nurses can better understand their new relationship with patients.

In addition to providing new training for nurses, it is important also to train front office staff in their new roles as they play a part in carrying out several PCMH principles, such as ensuring greater access and increased communication. Both clerical and clinical staff members are encouraged to develop a more sophisticated level of decision-making skills, which may require additional training on the PCMH principles, new workflow processes in the office, and increasingly integrated responsibilities to patients and each other. 

#### 3.3.2. Patient Experience

Every healthcare model must take into account patients' response to the new healthcare structure. Patients value the respect, quality, and concern of their healthcare provider in addition to accessibility; their reaction to the change in structure significantly affects the ability of a team to provide care [18]. It is equally important that patients understand that the ultimate goal of PCMH is to improve their quality of life and participate in achieving this objective. PCMH focuses not only on the manner in which care is administered, but also educating patients regarding their health and ensuring they have the knowledge necessary to improve it. National PCMH Demonstration Projects have shown patient education has increased their participation in their own care and subsequently their quality of life [7]. Patients are encouraged to interact with practice staff and their providers regularly during appointments and through new means of communication, such as online patient portals, and to take advantage of health education opportunities within the community to which they are referred by staff. 

### 3.4. Care Coordination

In PCMH, practices are tasked with improving care coordination and documenting patient engagement in self-management. This requirement has prompted many PCMHs to create a care manager or care coordinator position. This staff member (e.g., a nurse or nonclinical staff member) performs tasks that were previously delivered inconsistently or not at all, or that were formerly the responsibility of the physician, such as patient followups and patient education (When introducing the care coordinator to the patients, we encourage caution to avoid dramatization of past inadequacies in care delivery during patient and physician interactions, which could undermine their relationship with the physician [14].)

Throughout the PCMH conference, discussants emphasized their success with care coordinators in their practices, many of whom have received positive feedback from their patients. For instance, a diabetic patient from one of Carilion Clinic's Roanoke, Virginia practices reported

**I have recently been diagnosed with diabetes. While this was initially very upsetting for me, I was able to receive immediate counseling about my diabetes from the Care Coordinator. She took time with me one on one and that meant a lot. We reviewed some initial diet and exercise information, and then she set me up to see a dietitian in the Diabetes Management Program.**

This patient's improvement is unquestionable and shows the benefits this new position can provide. However, problems arise with the adoption of multidisciplinary team members in the education and support of patients, their time, salary, responsibilities, and workload are not defined in the cost or structure of a typical practice. Additionally, many practices that hire care coordinators face the challenge of reimbursing their work; this is because their work is typically not covered by health insurance at all or only to a limited extent.

In addition to self-management coaching of those patients with chronic conditions, the care coordinators at Carilion Clinic have been assigned the task of population management. The care coordinators use registries (registries are a list of patients with a chronic disease displayed with many clinical features to stratify level of disease control), to focus on specific chronic diseases, such as high blood pressure and diabetes. Care coordinators use predetermined protocols to contact those patients in poor disease control or those who are due for a follow-up visit. Once these patients come back into the office, a number of things happen as they reengage with their care team. The nurse updates the health maintenance care in the chart, the provider focuses on the acute or chronic disease issues, and the care coordinator offers to meet with the patient, further identifying barriers they face that may interfere with disease self-management. A sophisticated EMR or free-standing disease registry facilitates this task. These technologies are used to organize and target the entire patient panel to track health indicators and in turn, isolate the patients of interest. This disciplined approach to population management is not typical in most primary care offices; therefore, this activity tends not to be included in the budget, and time is not allocated for it. If the transitioning PCMH practice is committed to carrying out population management work, there needs to be added space for this individual to work along with a private consultation area to perform the self-management coaching. 

### 3.5. Staff Allocation and Clinical Space

Along with refocusing the office culture to adhere to the new comprehensive level of care, PCMH connects patients with resources in the healthcare system and the community. Primary care practices need to take time to build collaborative relationships with other professionals, such as social workers, nutritionists, and health educators, to provide needed services. It is not feasible for most PCMH practice sites to have space on hand for these professionals to work side by side at all times.

#### 3.5.1. Training and Transition Time

The PCMH model does not require that a specific staff member document the services offered, ordered, or rendered, and neither does NCQA. Rather, what is important is that there is a workflow process in place for such documentation in patients' medical records. For instance, at Carilion, ambulatory nurses have taken on roles in both chronic disease management and health maintenance. In some practices, nurses have taken on the documentation of all health maintenance needs of patients, while in others, this responsibility is shared among team members. No matter who plays what role, they need to be clearly within individual practice workflows and protocols. 

There may be a period when nurses will need to familiarize themselves with the new structure. During this time, temporary staffing can be useful to reduce backlogged work. For example, the Air Force experienced a backlog problem with the transition of nurses from floor nurses to disease managers. The disease manager started as one of the nurses in the clinic but was taken out of patient treatment and put into this new position. The number of nurses available to care teams was then cut in third. The problem is that this created backfill, so the disease management nurses were not able to concentrate on the task at hand but instead needed to address the problems that arose in the practice during their training. Once the Air Force's disease management nurses were able to focus on disease management, there was significant improvement in the quality of care for patients. It was also found in an outside study that patients who participated in chronic disease management had less frequent hospitalizations and on average spent 0.8 fewer nights in the hospital once admitted [2]. 

Carilion Clinic has also implemented disease management and has found that scanning the patient registry on a regular basis uncovers patients who would benefit from intensive disease management. In particular, one patient under Carilion's care, a 70-year-old woman with diabetes, coronary artery d status after coronary artery bypass grafting, and congestive heart failure, was oxygen dependent and wheelchair bound. She was contacted after the care coordinator saw in her EMR that she had visited the emergency room three times within the past eight months and one visit resulted in a prolonged hospital and Intensive Care Unit stay. Following the practice's protocol, her care team referred her to cardiac rehab. Subsequently, she has not visited the emergency room in over a year, has lost 30 pounds, no longer needs oxygen, and walks without assistance. 

#### 3.5.2. Resources for “Small” Practices

The size of the primary care practice can be an obstacle in implementing the PCMH model. Here, the term small is used to refer to primary care practices with only one or two physicians and an average-sized patient panel (It is not uncommon for a primary care practice with one physician to have a couple thousand patients; however, practices discussed within this paper do not have over 2,000 patients in their panel [19]). Approximately 32% of healthcare physicians practice in either an individual or a partnership practice, and 60% of practices function with 50 physicians or fewer [20]. Smaller practices typically cannot employ the same resources as larger facilities due to budget constraints, and therefore, they may have a harder time making the changes necessary to become a PCMH and have to find ways around their limited resources. For instance, rather than full-time coordinators in small practices, Carilion employs shared coordinators in sites where there are only one or two full-time providers; as such these smaller practices cannot rationalize adding a full-time staff member. National PCMH Demonstrations have shown that these smaller practices with such resource constraints have a more difficult time transforming to a high-functioning PCMH; they may qualify as Level 1, rather than Level 3, which means they only meet the minimum requirements of the NCQA [17–19]. An EMR is not required by the NCQA for a practice to apply for PCMH recognition, but having one may facilitate the practice's goals for care coordination. Implementing an EMR is not particularly difficult; instead, the concern is who will service, update, and sustain it. 

Regardless of the size of practices, PCMH principles encourage the use of electronic medical records to increase the efficiency of care coordination, although it is not required. This change in record keeping removes the need for medical record storage space within the practice and in turn allows for clinical space to be optimized.

#### 3.5.3. Facility Needs

Prior to fully transitioning to the PCMH model, healthcare organizations must address the issue of space. For instance, if a practice employs a care coordinator, they must be provided with space to communicate with patients in private, to perform their health assessments and individual disease coaching on the phone or in person, and to prepare plans for care, such as materials for the pre-visit huddle. It is uncommon for the traditional primary care practices to have a location for these types of private conversations and activities. Empty exam rooms or doctors' offices may just be sufficient, especially since these conversations taking place in waiting rooms or more public areas can easily violate confidentiality standards and the Health Insurance Portability and Accountability Act of 1996. 

#### 3.5.4. Integrating Electronic Medical Records

A fully integrated EMR helps PCMHs conduct team-based care for chronic conditions by allowing members to visualize the patient instantly, through the use of problem lists, immunization history, and medication lists. However, creating an EMR that administers the principles of PCMH has been a difficult task for primary care practices in transition [21]. In a 2006 study, it was reported that only 42% of primary care practices found it easy to generate a disease registry, while only 18% felt it was easy to create special follow-up appointments based on high-risk medications patients were taking [22]. Many of the PCMH Conference attendees had complaints about their difficulty in creating an EMR that suited their specific practice's needs. For practices transitioning from a paper-based system to an EMR, it takes staff members a great deal of time to input all of the information into a computerized system. Practices sometimes find the sheer volume of information needing to be recorded overwhelming and difficult to decipher, because there may not have been a PCMH template in paper charts. If the practice is unable to employ the EMR to its advantage, many of the goals of PCMH are very difficult to satisfy. For instance, care coordination and disease management are very difficult for large practices to handle using a paper-based system.

Once employed, EMRs can be used to improve patient care and therefore patient health by monitoring their health status to anticipate necessary treatments. Using the EMR, care coordinators and nurses are able to more efficiently search for specific patient types by viewing the patient's medical record on the computerized database and to acknowledge those who require immediate attention and contact them in a timely fashion. Staff then uses population management tools in the EMR to contact these individuals and encourage them to make an appointment [23]. In a review of the PCMH model, the authors were encouraged by PCMH physicians who are using proactive methods to assist unhealthy patients, rather than waiting for patients to seek help [24]. 

Carilion Clinic has created a structured method of administering disease management resulting in the optimization of their EMR. Their approach is to first search and then contact their patients who have a Priority One rating. Their searches are focused on patients with a chronic disease, which can be sorted in the EMR based on their conditions. Thus far, in their transition to a fully integrated EMR, Carillion Clinic has formed registries for asthma, congestive heart failure (CHF), diabetes (DM), and high blood pressure (HTN). Within each of the registries, the patient's clinical information, such as blood pressure, last appointment date, and glycohemoglobin (A1C) is recorded. Their registries can be sorted by the necessity of care or the priority as determined by Carillion Clinic. Priority One patients are those who have at least one of three predefined health conditions determined by Carilion Clinic (Carillion developed these predefined health conditions from nationally recognized medical guidelines). An example of a Priority One patient, as defined by Carilion Clinic, would be a diabetic (DM) with an A1C over 10. Another example of a Priority One patient would be a patient who is hypertensive, or a patient with a systolic BP over 160. The Carillion staff contacts patients with these conditions as soon as possible. Between the months of March and May 2010, of the diabetic patients and hypertensive patients contacted, 43% committed to an appointment with Carillion Clinic and their care team to address their current health status (As of June 11, 2010, 63% of the appointments made have been kept). Without a fully functioning EMR with capabilities that allow Carilion Clinic and other health care providers to search for these patients efficiently, they would remain invisible to the practice and may not receive the care they need. 

## 4. Conclusion: The Future of the PCMH Model

This paper has examined lessons learned from the implementation of PCMH at several sites, both military and civilian. PCMH demonstrations show there is still much work to be done, particularly through educating the medical community about the benefits of the model. Current PCMH demonstrations have shown that this method of care can improve the quality of life for patients, such as reducing the patient's frequency of ER visits and hospital admissions, as well as reducing health care costs [25, 26]. Overall, the results illustrate that PCMH decreases the gap between what providers say they want to do in order to improve patient care and what they can actually do with their current resources. 

As with any new healthcare structure, current PCMH practices are still developing methods to improve the quality of care provided to their patients, such as care coordination, team-based care, and the use of EMR to promote care coordination. There continues to be a struggle for transitioning PCMHs in determining how to implement the guidelines that the NCQA provides, which is a concern because NCQA is the most widely recognized organization in the United States for certifying practices as PCMHs [27]. NCQA recognition of PCMH does not require primary care practices to excel at every standard, but rather it requires a system of documentation about the quality of care being provided, and a plan for continuous improvement that follows each of the principles. However, this does not guarantee a successful practice; a successful practice should be defined as one that realizes PCMH recognition is not an end at all, but there is always room for improvement and maturation. With a great deal of enthusiasm from adopters thus far, PCMH can certainly have a permanent place in the future US healthcare system. 

Healthcare professionals and organizations will be under pressure to prove the “value” of their services. In implementing PCMH, one will track HbA1c, BMI, and/or other parameters as indicators of quality, but at what point would a PCMH be qualified to receive additional payment for their improvement in the quality of patient care? Without funding for additional resources, practices will continue with the discrepancy between the current volume-based healthcare system and a system that focuses on the quality of care. The question remains of whether PCMH is the direction healthcare should go in, and many studies show that preventative care improves the quality of life [28]. PCMH holds a great deal of potential in addressing the broken healthcare system in the United States, but at the same time, it also faces many obstacles in terms of payment reform, professional support, and patient participation.

## Figures and Tables

**Table 1:** Joint Principles of Medical Home*.

Personal physician	(i) Patients have an ongoing relationship with a personal physician (ii) First contact, continuous, and comprehensive care
Physician directed medical practice	(i) Personal physician leads a team of individuals at the practice level (ii) Collective responsibility for the ongoing care of patients
Whole-person orientation	(i) Medical home provides for all the patient's healthcare needs or appropriately arranges care with other qualified professionals (ii) Care for all stages of life: acute care, chronic care, preventive services, and end-of-life care
Care is coordinated and/or integrated	(i) Coordination of care across the healthcare system and patient's community (ii) Care is facilitated by registries, information technology, health information exchange, use of interpreters, and other means
Quality and safety	(i) Quality and safety improvement are hallmarks of the medical home (ii) Specific activities could include individualized care plans, evidence-based decision support tools, collection and reporting of quality improvement data, use of information technology, and voluntary certification of practices as medical homes
Enhanced access	(i) Patients can easily access healthcare via their medical home (ii) Specific improvements could include open access scheduling, expanded hours, and enhanced phone or e-mail communication
Payment	(i) Increased payments support the added level of service and value provided to patients who receive care from a medical home

*Stenger et al. [1].

**Table 2:** 2011 Revised NCQA Standards for medical home recognition*.

Enhance access and continuity	Identify and manage patient populations
Plan and manage care	Provide self-care support and community Resources
Track and coordinate care	Measure and improve performance

*NCQA [6].

## References

[1] Stenger RJ, DeVoe JE (2010). Policy challenges in building the Medical Home: do we have a shared blueprint?. *The Journal of the American Board of Family Medicine*.

[3] Lorig KR, Sobel DS, Stewart AL (1999). Evidence suggesting that a chronic disease self-management program can improve health status while reducing hospitalization a randomized trial. *Medical Care*.

[4] NCQA New NCQA Standards Take Patient-Centered Medical Homes to the Next Level.

[6] NCQA NCQA Patient-Centered Medical Home. http://ncqa.org/Portals/0/Programs/Recognition/2011PCMHbrochure_web.pdf.

[7] Nutting PA, Miller WL, Crabtree BF, Jaen CR, Stewart EE, Stange KC (2009). Initial lessons from the first national demonstration project on practice transformation to a patient-centered medical home. *Annals of Family Medicine*.

[8] Ginsburg PB, Maxfield M, O'Malley AS, Peikes D, Pham HH (2008). *Making PCMHs Work: Moving from Concept to Practice. Policy Prespective*.

[9] NCQA Physician Practice Connections- Patient-Centered Medical Home Standards and Guidelines. http://www.ncqa.org/tabid/629Default.aspx.

[10] Linzer M, Manwell LB, Williams ES (2009). Working conditions in primary care: physician reactions and care quality. *Annals of Internal Medicine*.

[11] Nutting PA, Crabtree BF, Miller WL, Stewart EE, Stange KC, Jaén CR (2010). Journey to the patient-centered medical home: a qualitative analysis of the experiences of practices in the National Demonstration Project. *Annals of Family Medicine*.

[12] Hsiao WC, Dunn DL, Verrilli DK (1993). Assessing the implementation of physician-payment reform. *The New England Journal of Medicine*.

[13] Rogers JC (2008). The patient-centered medical home movement—promise and peril for family medicine. *Journal of the American Board of Family Medicine*.

[14] Stewart EE, Nutting PA, Crabtree BF, Stange KC, Miller WL, Jaén CR (2010). Implementing the patient-centered medical home: observation and description of the national demonstration project. *Annals of Family Medicine*.

[15] Hughes SL, Weaver FM, Giobbie-Hurder A (2000). Effectiveness of team-managed home-based primary care: a randomized multicenter trial. *Journal of the American Medical Association*.

[16] Grumbach K, Bodenheimer T (2004). Can health care teams improve primary care practice?. *Journal of the American Medical Association*.

[17] Hansen M, Fisher JC (1998). Patient-centered teaching from theory to practice. *American Journal of Nursing*.

[18] Yedidia MJ (2007). Transforming doctor-patient relationships topromote patient-centered care: lessons from palliative care. *Journal of Pain and Symptom Management*.

[19] Friedberg MW, Safran DG, Coltin KL, Dresser M, Schneider EC (2009). Readiness for the patient-centered medical home: structural capabilities of massachusetts primary care practices. *Journal of General Internal Medicine*.

[20] Rittenhouse DR, Shortell SM (2009). The patient-centered medical home: will it stand the test of health reform?. *Journal of the American Medical Association*.

[21] Reid RJ, Fishman PA, Yu O (2009). Patient-centered medical home demonstration: a prospective, quasi-experimental, before and after evaluation. *The American Journal of Managed Care*.

[22] Audet AM, Davis K, Schoenbaum SC (2006). Adoption of patient-centered care practices by physicians: results from a national survey. *Archives of Internal Medicine*.

[23] Carnett WG (1999). Clinical practice guidelines: a tool to improve care. *Quality Management in Health Care*.

[24] Berenson RA, Hammons T, Gans DN (2008). A house is not a home: keeping patients at the center of practice redesign. *Health Affairs*.

[25] Flocke SA, Stange KC, Zyzanski SJ (1998). The association of attributes of primary care with the delivery of clinical preventive services. *Medical Care*.

[26] Ryan S, Riley A, Kang M, Starfield B (2001). The effects of regular source of care and health need on medical care use among rural adolescents. *Archives of Pediatrics and Adolescent Medicine*.

[27] Carrier E, Gourevitch MN, Shah NR (2009). PCMHs challenges in translating theory into practice. *Medical Care*.

[28] Lesser LI, Bazemore AW (2009). Improving the delivery of preventive services to medicare beneficiaries. *Journal of the American Medical Association*.

